# Adipocytes promote tumor progression and induce PD-L1 expression via TNF-α/IL-6 signaling

**DOI:** 10.1186/s12935-020-01269-w

**Published:** 2020-05-20

**Authors:** Zhi Li, Cai Zhang, Jing-Xia Du, Jia Zhao, Meng-Ting Shi, Man-Wen Jin, Hui Liu

**Affiliations:** 1 Department of Pharmacology, School of Basic Medicine, Tongji Medical College, Huazhong University of Science and Technology, Wuhan, China; 2 The Key Laboratory for Drug Target Researches and Pharmacodynamic Evaluation of Hubei Province, Wuhan, China

**Keywords:** Adipocytes, Hepatocellular carcinoma, Melanoma, PD-L1, TNF-α, IL-6

## Abstract

**Background:**

Obesity confers increased risk for various types of cancer. PD-L1 is a key molecule in tumor immune evasion by inducing T cell exhaustion. The relationship between obesity and PD-L1 is still ambiguous. This study was designed to reveal the development of hepatocellular carcinoma and melanoma in obese mice and to investigate if adipocytes regulate PD-L1 expression and the underlying mechanism.

**Methods:**

Monosodium glutamate-induced obese mice were inoculated with H22 tumor cells and High fat diet (HFD)-induced obese mice were inoculated with B16-F1 mouse melanoma cells. Human hepatoma HepG2 cells and B16-F1 cells were treated with conditional media from 3T3-L1 adipocytes (adi-CM). Neutralized anti-TNF-α and anti-IL-6 antibodies and inhibitor of NF-κB or STAT3 were used to reveal the mechanism of effect of adi-CM.

**Results:**

In obese mice, H22 and B16-F1 tumor tissues grew faster and PD-L1 expression in tumor tissue was increased. Adi-CM up-regulated PD-L1 level in HepG2 and B16-F1 cells in vitro. Differentiated 3T3-L1 adipocytes secreted TNF-α and IL-6, and neutralizing TNF-α and/or IL-6 reduced PD-L1 expression in adi-CM-treated cells. p-NF-κB/NF-κB level was downregulated in HepG2 and B16-F1 cells, and p-STAT3/STAT3 level was also decreased in HepG2 cells. In addition, inhibitor of NF-κB or STAT3 reversed the effect of adi-CM on PD-L1 expression.

**Conclusions:**

TNF-α and IL-6 secreted by adipocytes up-regulates PD-L1 in hepatoma and B16-F1 cells, which may be at least partially involved in the role of obesity in promoting tumor progression.

## Background

According to World Health Organization’s 2018 report, 39% of adults aged 18 years and over were overweight (BMI ≥ 25) in 2016, and 13% were obese (BMI ≥ 30) [1]. Overweight and obesity are associated with increased risk of liver cancer and malignant melanoma [2, 3]. During obesity, pro-inflammatory adipose tissue macrophages become more abundant and hundreds of adipokines secreted by adipose tissue may regulate cancer pathogenesis [4, 5]. Immune system continuously monitors cells and tissues including incipient cancer cells, overexpressed programmed death ligand 1 (PD-L1) in the tumor microenvironment engages programmed death 1 (PD-1) and subsequently triggers inhibitory signaling downstream of the T cell receptor [6]. Agents targeting PD-1/PD-L1, such as anti-PD-1 or anti-PD-L1 monoclonal antibody, displayed impressive antitumor effects in several malignancies and are now hailed as a great breakthrough in oncology [7, 8]. Although overweight could be considered a tumorigenic immune-dysfunction that could be effectively reversed by anti-PD-1/PD-L1 therapy [9], the relationship between adipose tissue and PD-1/PD-L1 is still ambiguous.

Cytokines are major regulators of adipose tissue metabolism. It has been shown that adipocytes can synthesize both Tumor necrosis factor (TNF-α) and several interleukins, notably IL-6 [10]. Negative impact of TNF-α on insulin sensitivity in obesity has been reported decades ago [11, 12]. The obesity-induced inflammatory microenvironment is a major drive of tumor progression, characterized by the presence of proinflammatory cytokines such as TNF-α [13, 14]. IL-6 is an important signaling molecule to affect immune system, lipid metabolism, insulin resistance, mitochondrial activity [15–17], and also promotes hepatocellular carcinoma (HCC) and melanoma progression [18, 19]. Recently, TNF-α and IL-6 have also been found as regulators of PD-L1 in a variety of cells [20, 21], however, whether they can regulate PD-L1 expression in HCC or melanoma is unknown.

Nuclear factor κB (NF-κB) is a family of transcription factors and can be activated by TNF-α receptor [22]. NF-κB contributes to oncogenesis in a majority of cell types including HCC [23]. Similarly, STAT3, a transcription factor, acts downstream of various cytokines and is found to be constitutively active in a variety of cancers [24]. A recent study demonstrated increased hepatic pSTAT3 level in obese mice and human and identified STAT3 as a driver of HCC progression [25]. More importantly, NF-κB and STAT3 are reported to be key mediators of PD-L1 [26–29].

Based on those findings above, the present study was designed to reveal the development of hepatocellular carcinoma in obese mice and to clarify if adipocytes regulate PD-L1 expression and the underlying mechanism.

## Methods

### Animal experiments

Male CD1 mice were used to establish monosodium glutamate-induced obese (MSG-IO) model as prior research of our lab [30]. Male C57BL/6J mice were fed with High fat diet (HFD, D12492, 60 kcal %) to establish diet-induced obese (DIO) model. All experiments were approved by the Ethics Committee of Animal Use for Teaching and Research of Tongji Medical College at Huazhong University of Science and Technology and were in accordance with the guide for the care and use of laboratory animals of the US National Institutes of Health Guidelines. All mice were housed in a temperature (22 ± 2 °C) and humidity controlled (50 ± 5%) room with a cycle of 12 h light/12 h darkness and free access to food and water. At the age of 15 weeks, control and MSG-IO mice were inoculated with 1 × 10^5^ H22 mouse hepatoma cells at right armpit. After 17 days of inoculation, all mice were sacrificed, and tumor tissues were dissected and weighed. As for C57BL/6J mice, after fed with HFD or normal chow diet (NCD) for 24 weeks, 1 × 10^5^ B16-F1 mouse melanoma cells were injected at right armpit. After 20 days of inoculation, all mice were sacrificed, and tumor tissues were dissected and weighed. Before the inoculation, body weight, body length (from anus to nose), waist circumference and Lee’s index were measured as estimates of body fat. Lee’s index was calculated as bodyweight (g)^1/3^/body length (cm)*1000.

### Cell culture and Differentiation of 3T3-L1 cells

H22, B16-F1, human hepatoma HepG2 cells and 3T3-L1 preadipocytes were purchased from China Center for Type Culture Collection (Wuhan, China). The cells were cultured in DMEM media containing penicillin/streptomycin (100 U/100 μg/ml) and 10% fetal bovine serum, and incubated at 37 °C/5% CO_2_.

3T3-L1 preadipocytes were differentiated through incubation in complete DMEM containing 10 μg/mL insulin, 0.5 μM dexamethasone, and 0.5 mM 3-isobutyl-1-methylxanthine (IBMX) for 2 days and thereafter in complete DMEM supplemented with 10 μg/mL insulin for next 2 days. Subsequently, cells were maintained in and re-fed every 2 days with culture medium for another 4 days. Differentiation into mature adipocytes was confirmed by Oil Red O staining. Differentiated 3T3-L1 cells were cultured with complete DMEM for 24 h, and supernatants were collected as adipocytes conditional media (adi-CM).

HepG2 or B16-F1 cells were treated with adi-CM for 48 h. In the meantime, anti-TNF-α antibody (R&D system, AF-410-SP) and/or anti-IL-6 antibody (R&D system, MAB406), the inhibitor of STAT3 BP-1-102 (Selleck, S7769) or the inhibitor of NF-κB withaferin A (MCE, HY-N2065) were used at corresponding concentrations.

### Oil red O staining of adipocytes

3T3-L1 adipocytes and preadipocytes were stained with oil red O (Servicebio) for 15 min and washed with PBS. The positively stained cells were captured under the microscope. To measure the lipid accumulation, Oil red O stained cells were washed with isopropyl alcohol and supernatants were tested by Multiscan Spectrum at 492 nm.

### Immunohistochemistry

Tumor tissues were fixed in formalin and processed for paraffin embedding. Tissue sections of 5-μm thickness were cut and de-waxed and hydrated through graded ethanols, cooked in 25 mM citrate buffer pH 6.0 (115 °C, 3 min), transferred into boiling deinoized water and left to cool down for 20 min. The sections were then stained with anti-CD8 antibody (Abcom, GB11068) at 4 °C overnight, washed three times with PBS and stained with HRP (Servicebio, G23303) for 1 h. Positive area will be stained in brown with DAB developer.

### Western blotting and ELISA

Tumor tissues or cells were lysed in RIPA buffer with a protease inhibitor cocktail. Protein was separated by SDS-PAGE gel and transferred to PVDF membrane (Millipore) for 90 min. The membrane was blocked for 60 min in 5% skim milk at room temperature. The membrane was briefly rinsed with 1xTBST and incubated with the respective primary antibodies at 4 °C overnight. Primary antibody of PD-L1 was purchased from NOVUS (NBP1-76769), and p-STAT3 (#9145), STAT3 (#9139), p-NF-κB (#3033), NF-κB (#8242), β-actin (#3700) and GAPDH (#5174) were purchased from CST. After incubation with the secondary antibodies, the protein bands were developed with the chemiluminescent reagents.

Media from adipocytes were collected and TNF-α and IL-6 levels were measured by using ELISA kits (Multi Sciences, EK282, EK260).

### Statistical analysis

Experimental data are shown as mean ± SEM. Statistical analysis was performed with unpaired Student’s *t* test, or one-way ANOVA with Newman-Keuls. Differences were considered statistically significant at *P *< 0.05.

## Results

### MSG-IO and DIO mice exhibit obvious obesity and promoted tumor growth

**Fig. 1:**
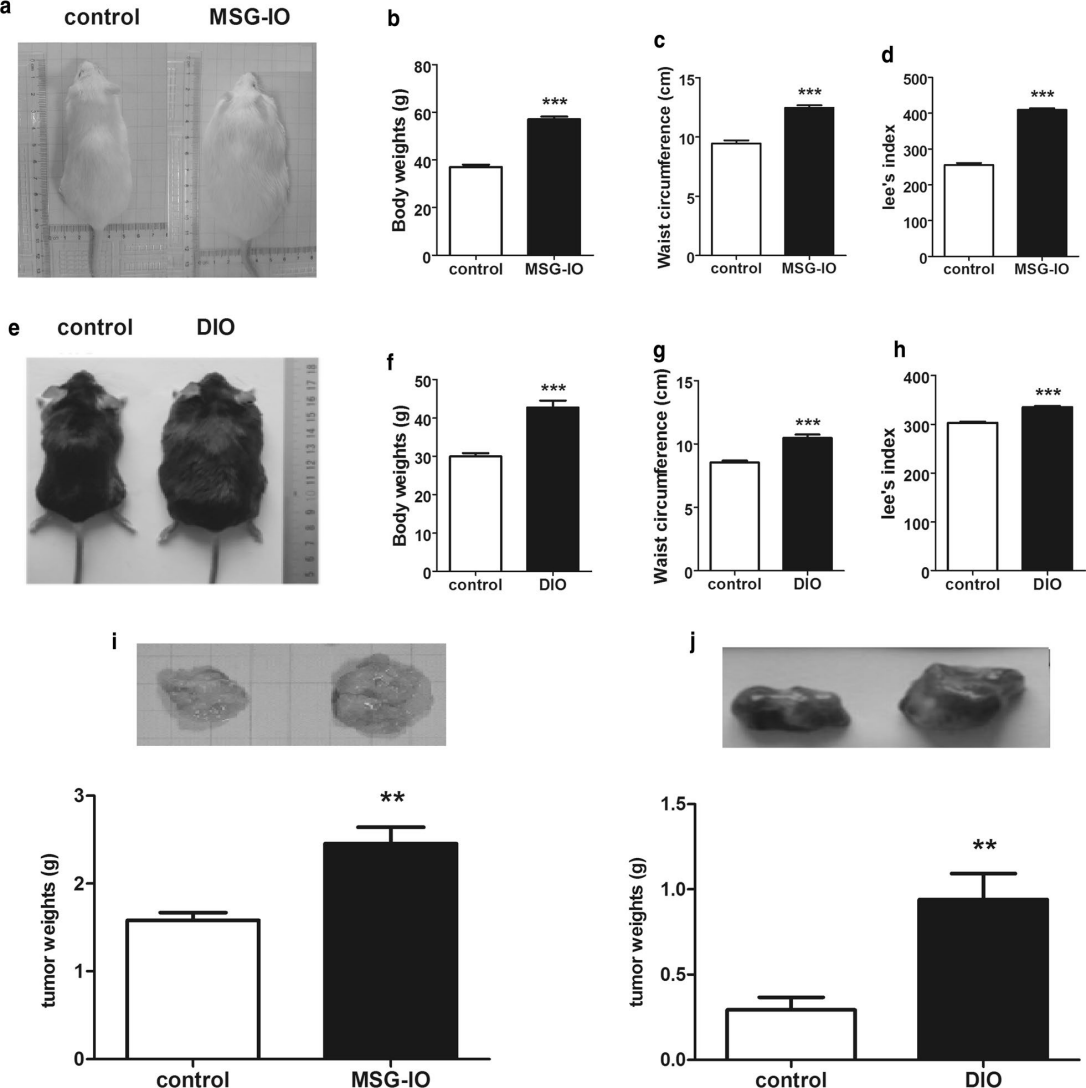
Tumor growth was promoted in MSG-IO and DIO mice. **a** Representative images of control and MSG-IO mice at 15 weeks of age. **b** Body weight, waist circumference (**c**) and Lee’s index (**d**) measured in MSG-IO model. **e** Representative images of control and DIO mice at 24 weeks of age. **f** Body weight, waist circumference (**g**) and Lee’s index (**h**) measured in DIO model. **i** Representative images and weights of tumor tissues in MSG-IO model after 17 days of cell inoculation. **j** Representative images and weights of tumor tissues in DIO model after 20 days of cell inoculation. Data are expressed as mean ± SEM, n = 12, **P < 0.01 and ***P < 0.001 Vs control

MSG-IO and DIO mice presented significant fat phenomenon and were used in our experiment to study tumor progression in obesity individuals. Before the incubation, body weights, waist circumference and Lee’s index were all significantly increased in MSG-IO mice (Fig. 1a–d) and DIO mice (Fig. 1e–h). 10^5^ H22 hepatoma cells within 0.2 ml of 0.9% saline were injected into control and MSG-IO mice. 17 days later, mice were sacrificed and tumor tissues were carefully dissected. H22 tumor tissue grew faster in MSG-IO mice (Fig. 1i). Similarly, 20 days after the injection of 10^5^ B16-F1 cells in control and DIO mice, weights of B16-F1 tumor tissue were also increased in obese mice (Fig. 1j). These results indicated that tumor proliferation was accelerated in obese mice.

### Tumor PD-L1 expression is increased in obese mice

**Fig. 2:**
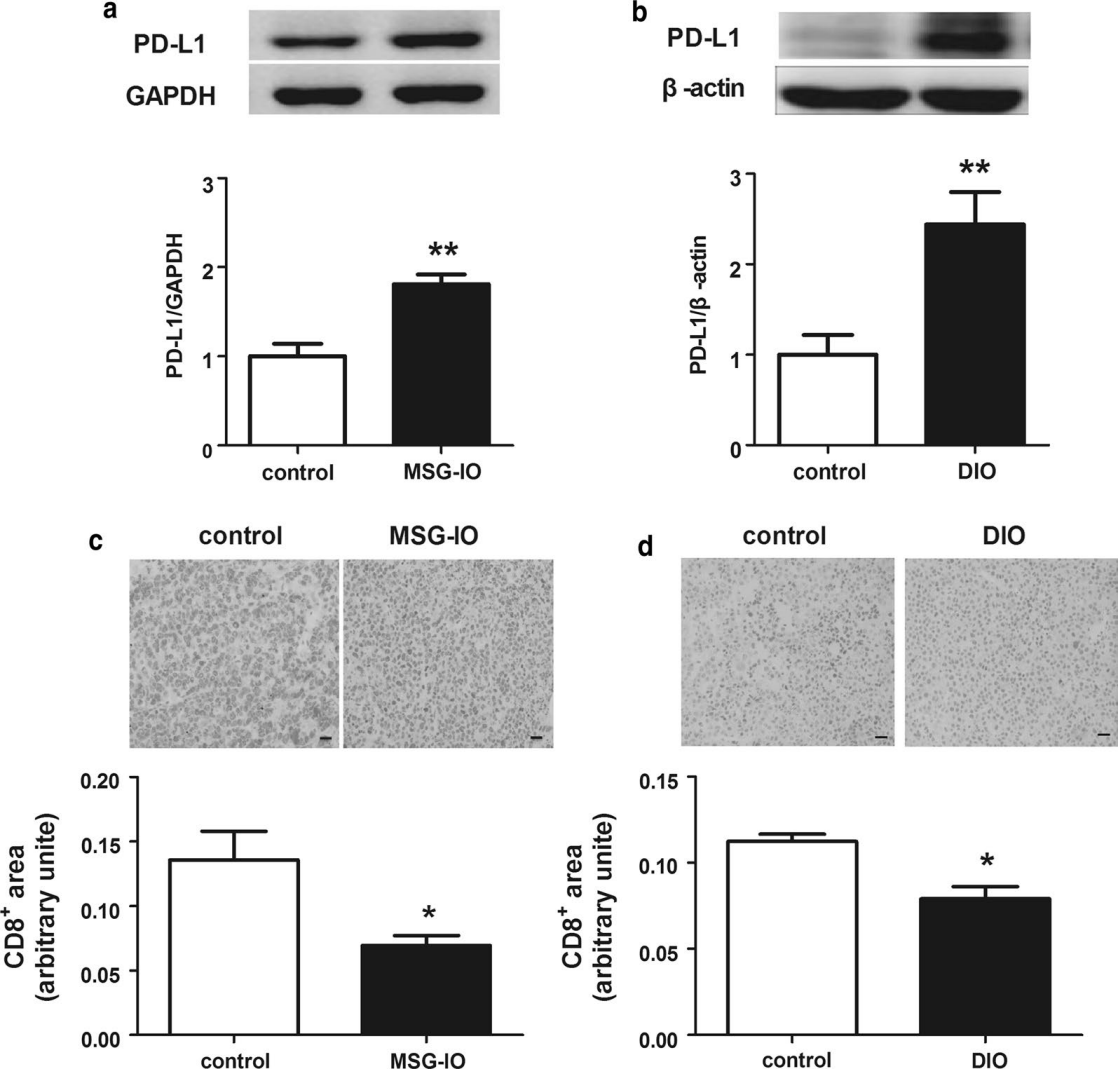
PD-L1 expression of tumor tissue was increased in obese mice. **a** PD-L1 protein levels in tumor tissue of mice in MSG-IO model detected by western blot. **b** PD-L1 protein levels in tumor tissue of mice in DIO model detected by western blot. **c** Representative immunohistochemistry staining and quantitative analysis of CD8^+^ T cells in H22 tumor tissue. **d** Representative immunohistochemistry staining and quantitative analysis of CD8^+^ T cells in B16-F1 tumor tissue. Scale bar 50 μM. Data are expressed as mean ± SEM, n = 6 (western blot) and n = 3 (immunohistochemistry), *P < 0.05 and **P < 0.01 Vs control

PD-1/PD-L1 pathway is a key regulator in tumor immune evasion. We next checked the PD-L1 protein level in tumor tissue in control and obese mice. PD-L1 expression was elevated in tumor tissues of obese mice (Fig. 2a, b), and we found that CD8^+^ T cells were decreased in obese mice tumor tissue (Fig. 2c, d). It suggested that activation of PD-1/PD-L1 pathway induced the exhaustion of tumor infiltrating lymphocytes (TIL). These data illustrated that tumor PD-L1 expression is boosted in obese state, thus, TIL filtration is inhibited and an immune evasive microenvironment is provided.

### 3T3-L1 adipocytes conditional media increases PD-L1 expression

**Fig. 3:**
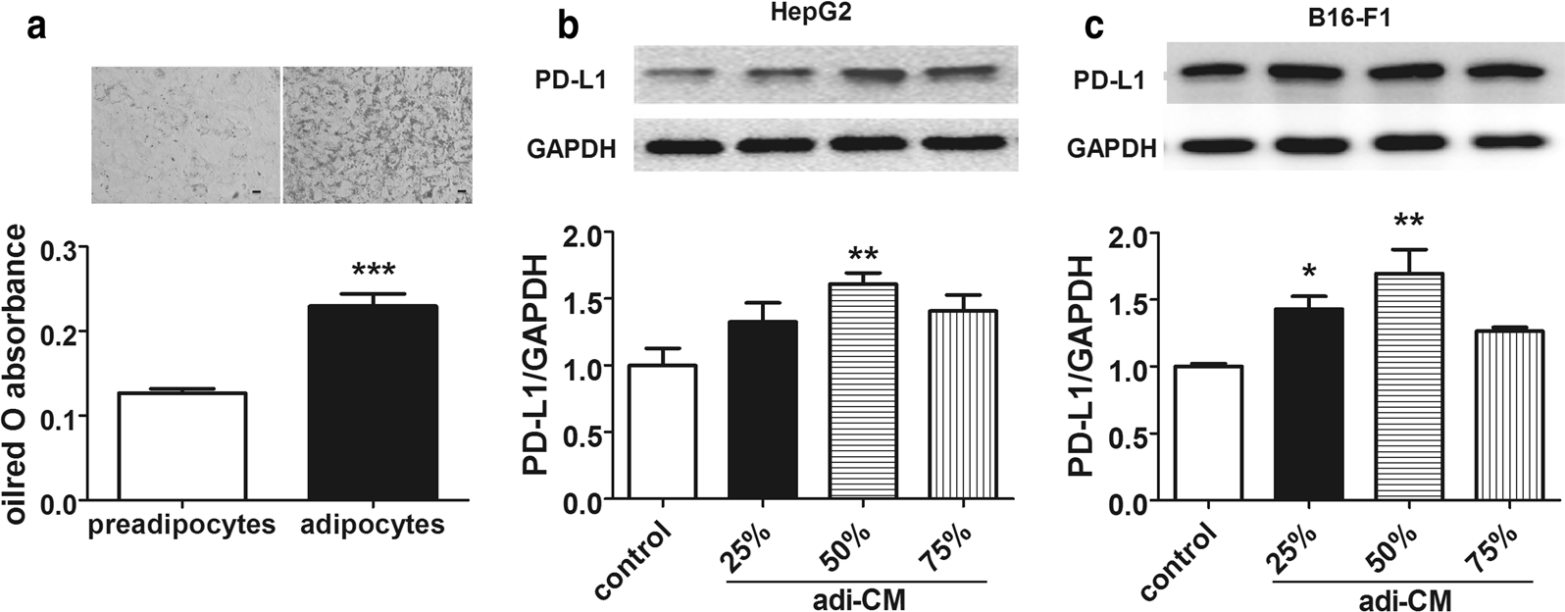
Conditional media of 3T3-L1 adipocytes induced PD-L1 expression on HepG2 and B16-F1 cells. **a** Oil-red O staining and quantification of lipids in 3T3-L1 preadipocytes and adipocytes. Scale bar 10 μm. Data are expressed as mean ± SEM, n = 9, ***P < 0.001 Vs preadipocytes group. **b** PD-L1 levels of HepG2 cells treated with or without adipocytes conditional media (adi-CM) for 48 h. **c** PD-L1 levels of B16-F1 cells treated with or without adi-CM for 48 h. Data are expressed as mean ± SEM, n = 6, *P < 0.05 and **P < 0.01 Vs control

We next investigated the possible mechanism of elevation of PD-L1 expression in obese state. In obesity, the enlargement of the white adipose tissue (WAT) releases free fatty acids and inflammatory cytokines. To imitate the function of WAT, we used 3T3-L1 adipocytes conditional media in cells experiment. 3T3-L1 preadipocytes were differentiated to adipocytes and presented an apparent content of lipids (Fig. 3a). After differentiation, 3T3-L1 adipocytes were cultured with fresh media for 24 h and supernatant were collected as adi-CM. HepG2 and B16-F1 cells were treated with different proportions of adi-CM for 48 h, and PD-L1 protein was detected, respectively. It showed that 50% of adi-CM presented the most effect on PD-L1 expression (Fig. 3b, c). In the subsequent test, 50% of adi-CM was set to induce the model of up-regulating PD-L1. This result implied that some factors secreted by adipocytes could activate PD-L1 in HCC and melanoma cells.

### Blockade of TNF-α/IL-6 downregulates PD-L1 expression in adi-CM-treated tumor cells

**Fig. 4:**
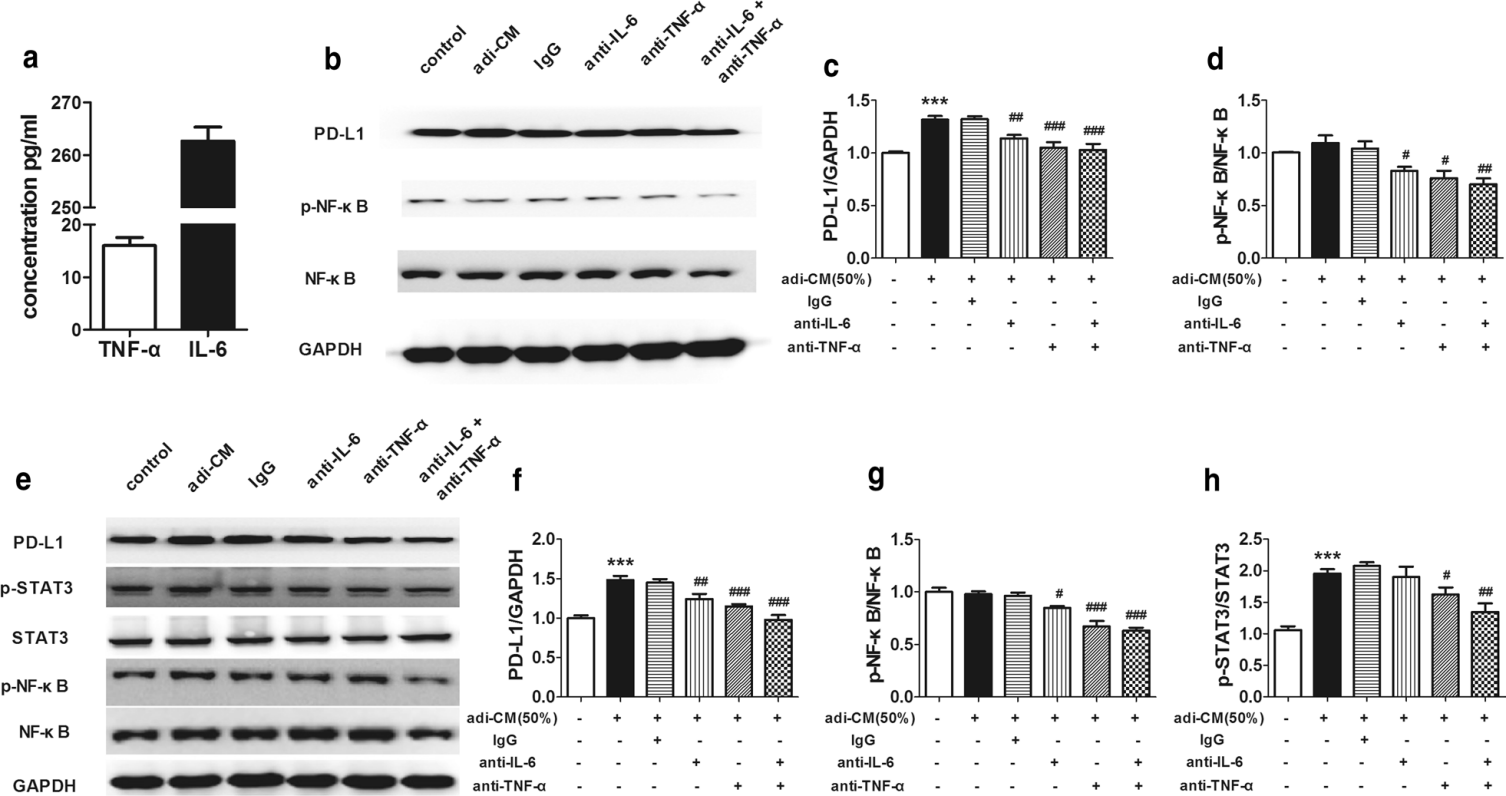
Blockade of TNF-α/IL-6 decreased PD-L1 expression. **a** Levels of TNF-α and IL-6 in the supernatant of differentiated 3T3-L1 adipocytes detected by ELISA. **b** Representative western blot images of PD-L1 and phosphorylated-NF-κB protein expression in adi-CM-treated B16-F1 cells. Anti-TNF-α antibody and/or anti-IL-6 antibody were used in neutralizing TNF-α and IL-6. **c** Quantification of PD-L1 and phosphorylated-NF-κB (**d**) protein expression on B16-F1 cells treated. **e** Representative western blot images of PD-L1, phosphorylated-STAT3 and phosphorylated-NF-κB protein expression in adi-CM-treated HepG2 cells with the addition of anti-TNF-α antibody and/or anti-IL-6 antibody. **f** Quantification of PD-L1, phosphorylated-NF-κB (**g**) and phosphorylated-STAT3 (**h**) protein expression on HepG2 cells treated. Data are expressed as mean ± SEM, n = 4 (ELISA) and n = 6 (western blot), ***P < 0.001 Vs adi-CM group. ^#^*P *< 0.05, ^##^*P *< 0.01 and ^###^*P *< 0.001 Vs IgG group

Either TNF-α or IL-6 has been found as a regulator of PD-L1 in some cell types, thus, we test the ability of 3T3-L1 adipocytes to produce TNF-α and IL-6. The results showed that 3T3-L1 adipocytes did secrete TNF-α and IL-6 (Fig. 4a). To test whether TNF-α or IL-6 mediates PD-L1 expression in adi-CM model, mouse anti-TNF-α antibody (100 ng/ml) and/or mouse anti-IL-6 antibody (100 ng/ml) were used to neutralize certain factors secreted from adipocytes. After 48 h of incubation, proteins were detected. We found that inhibition of TNF-α/IL-6 downregulated PD-L1 expression and reduced p-NF-κB/NF-κB level in B16-F1 cells (Fig. 4b–d). Similar results were found in HepG2 cells experiments (Fig. 4e–h). PD-L1 and p-NF-κB/NF-κB protein expression of HepG2 cells were decreased after the addition of anti-TNF-α and/or anti-IL-6 antibody. Inhibition of TNF-α also reduced p-STAT3/STAT3 level. Neutralizing IL-6 had no significant effect on p-STAT3/STAT3 expression, but the combination of two antibodies showed a greater effect than the use of any single antibody. These results indicated that TNF-α and IL-6 derived from adipocytes induced PD-L1 expression in HepG2 and B16-F1 cells.

### Inhibition of NF-κB or STAT3 effectively reverses the adi-CM effect

**Fig. 5:**
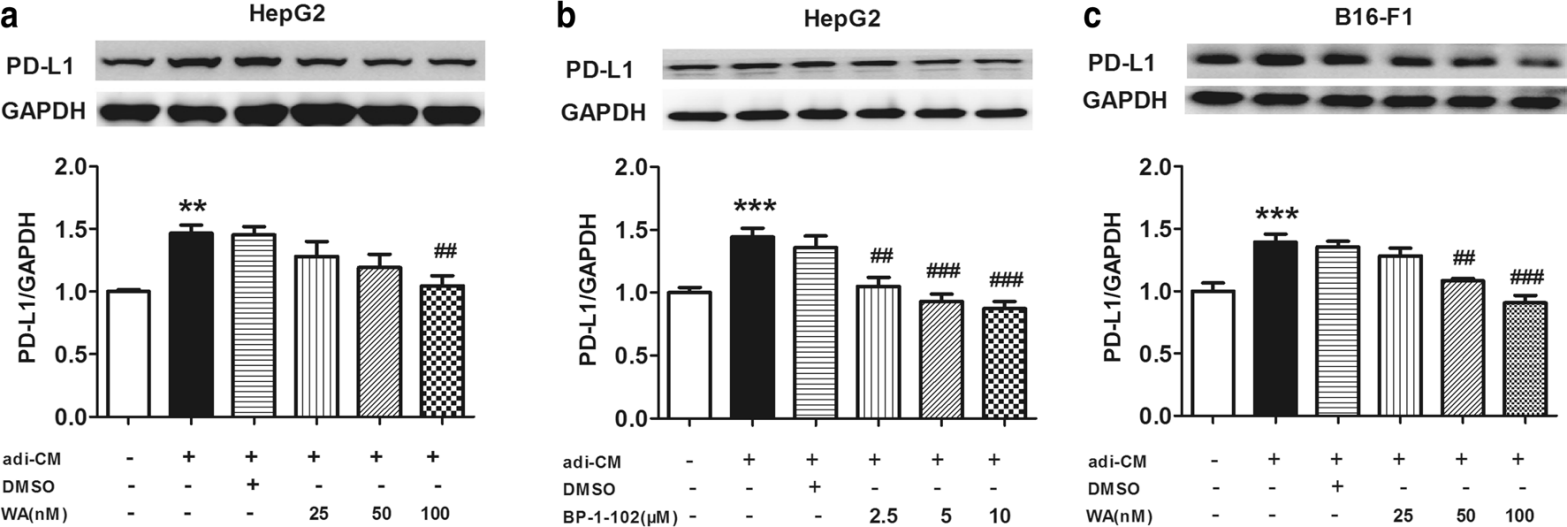
Inhibition of NF-κB or STAT3 downregulated PD-L1 expression in adi-CM model. **a** PD-L1 protein levels in HepG2 cells treated with different concentration of NF-κB inhibitor, withaferin A (WA). **b** PD-L1 protein levels in HepG2 cells treated with different concentration of STAT3 inhibitor, BP-1-102. **c** PD-L1 protein levels in B16-F1 cells incubated with WA. Data are expressed as mean ± SEM, n = 6, **P < 0.01, ***P < 0.001 Vs adi-CM group. ^##^*P *< 0.01 and ^###^*P *< 0.001 Vs DMSO group

To further investigate whether NF-κB or STAT3 inhibition influence PD-L1 expression, different concentration of NF-κB inhibitor, withaferin A (WA) and STAT3 inhibitor, BP-1-102 were used to treat HepG2 cells in adi-CM model, respectively. 100 nM WA apparently inhibited PD-L1 expression (Fig. 5a), and PD-L1 level was reduced by BP-1-102 in a concentration-dependent manner (Fig. 5b). PD-L1 expression of adi-CM-treated B16-F1 was also decreased by WA (Fig. 5c). The effect of the inhibitors on PD-L1 expression suggests that NF-κB or STAT3 is a key mediator of PD-L1.

## Discussion

There are strong epidemiological associations between fat mass and the incidence of a variety of malignancies, and obesity is now considered a major modifiable risk factor for cancer [31]. Both MSG-IO and DIO mouse models are commonly used models in obesity research [32, 33]. MSG obesity is an early-onset obesity resulting from MSG-induced lesions in arcuate nucleus to neonatal mice, further disrupting the hypothalamic signaling cascade of leptin action. DIO obesity is a late-onset obesity, it developed during 16-week-long feeding with high-fat diet containing high calories as fat. In the present study, these two models are chosen to help us identify the association between obesity and cancer.

In recent years, it has become evident that obesity is involved in HCC progression in many ways, including elevated proinflammatory cytokines induced by oxidative and endoplasmic reticulum stress, dysregulation of adipokines, altered gut microbiota and extracellular vesicles [34–37]. The association of adiposity with melanoma is not as strong as liver cancer [38], and the mechanism of obesity promoting melanoma progression is little known. We found that either H22 tumor tissue or B16-F1 tumor tissue did grow faster in different obese mouse model, which confirmed the tumor-promoting effect of obesity.

The immune system can eliminate tumor cells and protect against chronic inflammatory environment that promotes cancer [39]. Cytotoxic T cells are essential for targeting and destroying infected or tumorigenic cells. PD-1 and its ligand PD-L1, a pair of checkpoint molecules, induce T cell exhaustion. Importantly, PD-L1 is expressed in peripheral tissues and most cancer cells, a high level PD-L1 environment may help cancer cells avoid from T cell attack. Although obesity has become a predominant risk factor for cancer, little is known about the relationship between PD-L1 and obesity. We found that PD-L1 expression in H22 tumor tissue was increased and TIL was exhausted in MSG-IO mice. It implies that upregulation of PD-L1 may be involved in the tumor-promoting effect of obesity. The similar result was found in DIO mice, B16-F1 cells exhibited elevated PD-L1 expression, and our results may provide one possibility that would explain the increasing risk of melanoma in obese individuals.

We then further investigated the mechanism of elevation in PD-L1 expression in obese state. We demonstrated that CM of 3T3-L1 adipocytes induced increase in PD-L1 protein level in HepG2 and B16-F1 cells. 50% of adi-CM significantly increased PD-L1 expression of HepG2 and B16-F1 cells. With a higher proportion (75%) of adi-CM, the effect on PD-L1 reduced. This may be due to the lack of serum and other nutritive content. Hundreds of factors can be secreted by adipocytes [40], and we focused on TNF-α and IL-6, which have been reported to regulate PD-L1 on kinds of cancer cells but HCC or melanoma. We first detected the amount of TNF-α or IL-6 in adi-CM. The results showed that 3T3-L1 adipocytes did secrete TNF-α and IL-6, and the amount of TNF-α or IL-6 is consistent with that reported by Jung et al. and Zieger et al. respectively [41, 42]. Furthermore, we proved that anti-TNF-α and anti-IL-6 antibody could reverse the effect of adi-CM, which means blockade of TNF-α or IL-6 could downregulate PD-L1 expression. These results suggest that TNF-α and IL-6 signaling mediates PD-L1 expression, therefore involved in tumor progression in obese state. Other adipokines, such as IL-17 or HIF-1α, which have been reported as a regulator of PD-L1 [43, 44], may also play an important role, and further research would be needed.

We also detected the expression of NF-κB and STAT3, the most common downstream factors of TNF-α and IL-6, respectively. p-NF-κB/NF-κB levels were decreased as expected in both HepG2 cells and B16-F1 cells treated. TNF-α is also an activator of STAT3 [45], in line with this, we found p-STAT3 level was reduced when TNF-α was neutralized in HepG2 cells. Interestingly, anti-IL-6 treatment seems to have no influence on STAT3 activation, but combination of two antibodies exhibited a stronger inhibition than use of anti-TNF-α alone. It may indicate that TNF-α and IL-6 had synergistic effect on regulating STAT3 activation in HCC. In addition, the use of NF-κB or STAT3 inhibitor decreased PD-L1 expression in adi-CM-treated tumor cells, which provided more evidence that TNF-α and/or IL-6 signaling mediated the regulation of PD-L1 in adi-CM-treated tumor cells. It is a pity that the protein expression of p-STAT3 and STAT3 in B16-F1 cells is not included in the results. Several attempts were made to detect the expression levels of p-STAT3 and STAT3 in B16-F1 cells but failed.

## Conclusions

The present study, for the first time, demonstrated that TNF-α and IL-6 secreted by adipocytes could upregulate PD-L1 level in HCC and melanoma. This may be partially involved in the role of obesity in promoting tumor progression. Considering that WAT accounts for a certain proportion in obese individuals, targeting WAT may improve metabolic environment and reduce tumorigenesis.

## Data Availability

All the data used to support the findings of this study are available from the corresponding author upon request.
